# Sequences for reporting on mild and serious types of suicidal behaviours: A population-based study in Latvia in 2010-2018

**DOI:** 10.1192/j.eurpsy.2022.2170

**Published:** 2022-09-01

**Authors:** K. Mieze, A. Kivite-Urtane, D. Grinberga, B. Velika, I. Pudule, E. Rancans

**Affiliations:** 1 Riga Stradins University, Department Of Doctoral Studies, Riga, Latvia; 2 Riga Stradins University, Department Of Public Health And Epidemiology, Riga, Latvia; 3 Centre for Disease Prevention and Control of Latvia, Department Of Research And Health Statistics, Riga, Latvia; 4 Riga Stradins University, Department Of Psychiatry And Narcology, Riga, Latvia

**Keywords:** Suicide, suicide prevention, suicidal behaviour, public health

## Abstract

**Introduction:**

Latvia is listed as a country with one of the highest suicide mortality rates in European Union (National Statistical System of Latvia, 2021).

**Objectives:**

To assess the sequences for reporting of suicidal behaviours (SB) in Latvian general population.

**Methods:**

The study is based on secondary data of the Health Behaviour Among Latvian Adult Population survey, provided by the Centre for Disease Prevention and Control of Latvia (representative sample of the Latvian population aged 15-64 in 2010, 2012, 2014; 15-74 in 2016, 2018; (n=16,105). Respondents were asked to report the occurrence of life-weariness (LW), death wishes (DW), suicidal ideation (SI), suicidal plans (SP), suicide attempts (SA) during the previous year.

**Results:**

Statistically significant differences between genders were found for LW (Χ²=17.118; df=1; *p*<0.001; AR=±4.1), DW (Χ²=17.764; df=1; *p*<0.001; AR=±3.8) and any type of SB (Χ²=15.721; df=1; *p*<0.001; AR=±3.8). Frequencies of individual sequences of reporting last year SB are presented in Table.

**Table:** 

Sequences for reporting on SB in 2010-2018		
	N	%
**Continious**		
LW	885	35.3
LW+DW	746	29.7
LW+DW+SI	255	10.2
LW+DW+SI+SP	300	12.0
LW+DW+SI+SP+SA	51	2.0
**Continious not complete**		
DW	146	5.8
DW+SI	13	0.5
**Non-continious**		
LW+DW+SI+SA	0	0.0
LW+DW+SP	13	0.5
LW+SI	44	1.8
LW+SI+SP	7	0.3
LW+SI+SP+SA	1	0.0
LW+SP	8	0.3
DW+SI+SA	0	0.0
DW+SP	0	0.0
SI	16	0.6
SI+SA	0	0.0
SI+SP	3	0.1
SP	13	0.5
Other	9	0.4
**Total**	2510	100

**Conclusions:**

Further research is warranted to identify vulnerable groups in the Latvian general population in relation to suicidality and thus to develop targeted preventive measures.

**Disclosure:**

This work has been developed with financing from the European Social Fund and Latvian state budget within the project no. 8.2.2.0/20/I/004 “Support for involving doctoral students in scientific research and studies at Rīga Stradiņš University.

